# Facile Synthesis of Colored and Conducting CuSCN Composite Coated with CuS Nanoparticles

**DOI:** 10.1186/s11671-017-2275-6

**Published:** 2017-08-23

**Authors:** E. V. A. Premalal, Yasun Y. Kannangara, S. P. Ratnayake, K. M. Nalin de Silva

**Affiliations:** 1 Sri Lanka Institute of Nanotechnology (SLINTEC), Nanotechnology & Science Park, Mahenwatta, Pitipana, Homagama, Sri Lanka; 2 Department of Chemistry, University of Colombo, Colombo 03, Sri Lanka

**Keywords:** CuS nanocoating, Colored conducting composite, CuSCN, IR absorption

## Abstract

Conductivity-tunable, different colored CuS nanoparticle-coated CuSCN composites were synthesized in a single pot using a mixture of copper sulfate and sodium thiosulfate in the presence of triethyl amine hydrothiocyanate (THT) at the ambient condition. When these reagents are mixed in 1:1:1 molar ratio, white-gray-colored CuSCN was produced. In the absence of THT, microsized dark blue-colored CuS particles were produced. However, when THT is present in the solution mixture by different amounts, colored conducting CuS nanoparticle-coated CuSCN composite was produced. CuS nanoparticles are not deposited on CuSCN soon after mixing these regents, but it takes nearly overnight to see the color change (CuS production) in the white CuSCN dispersed mixture. TEM analysis shows that composite consists of hexagonal CuS nanoparticles in the range of ~ 3–10 nm in size. It is interesting to note that CuS-coated CuSCN possesses higher conductivity than neat CuS or CuSCN. Moreover, strong IR absorption was observed for CuS-coated CuSCN composite compared to neat CuS (absence of THT) or CuSCN. Lowest resistivity of 0.05 Ω cm was observed for annealed (250 °C) CuS-coated CuSCN particles (adding 10 ml of THT) under nitrogen atmosphere. Also, this simple method could be extended to be used in the synthesis of CuS-coated composites on the other nanomaterials such as metal oxides, polymers, and metal nanoparticles.

**Electronic supplementary material:**

The online version of this article (doi:10.1186/s11671-017-2275-6) contains supplementary material, which is available to authorized users.

## Background

Synthesis of nanostructured materials has attracted much attention due to their unique optical, electrical, mechanical, and electronic properties which cannot be obtained from macroscopic materials. Copper sulfide has drawn significant interest owing to the variations in stoichiometric composition, valence states, nanocrystal morphologies, complex structures, and their different unique properties [1–5]. The stoichiometric composition of copper sulfide varies in a wide range from Cu_2_S at copper-rich side to CuS_2_ at the copper-deficient side, such as CuS, Cu_1.96_S, Cu_1.94_S, Cu_1.8_S, Cu_7_S_4_, and Cu_2_S [6, 7]. In the copper-rich section, all the stable compounds of Cu_*x*_S are p-type semiconductor as the copper vacancies are within the lattice [8]. As a p-type semiconductor with small bandgap and high ionic conduction, Cu_*x*_S nanocrystals are expected to be notable candidates for photovoltaics, field emission devices, and lithium-ion batteries [9–11].

CuS (covellite) shows excellent metallic conductivity, and it is possible to transform this to type 1 superconductor at 1.6 K [12]. It has attracted utilizability in several potential applications such as in photocatalysis [13], photovoltaics [9], cathode materials [14], supercapacitors [15], and lithium ion batteries [11]. Various morphologies of CuS such as nanowires [16], nanodisks [17], hollow spheres [18], and flower-like structures [19] have been reported by using different preparation methods, mostly by hydrothermal method.

Several studies on CuS-based composite are reported [20–30]. Yuan et al. have synthesized CuS (nanoflower)/rGo composite using ultrafast microwave-assisted hydrothermal method using Cu(NO_3_)_2_ and thiourea for lithium storage application [21]. Yu et al. have synthesized CuS/ZnS nanocomposite hollow spheres with diameters of about 255 nm and shells composed of nanoparticles by an ion-exchange method using monodisperse ZnS solid spheres as a precursor [22]. Hong et al. have synthesized CuS-coated ZnO rod by two-step dipping methods in the sodium sulfide and copper sulfate for piezo-photocatalytic application [23]. Bagheri et al. have synthesized CuS-coated activated carbon by mixing of activated carbon in the mixture of copper(II) acetate and thioacetamide for the removal of ternary dyes [24].

In the present study, we have synthesized CuS nanoparticle-coated different colored CuSCN composites employing a mixture of copper sulfate and sodium thiosulfate in the presence of triethyl amine hydrothiocyanate (THT) at the ambient condition. This method enables us to produce different colored and conductivity-tunable CuS-coated CuSCN particles. This composite shows excellent optical and electrical properties as explained below. Here, we have selected CuSCN, p-type, high-bandgap (~ 3.6 eV), and air-stable semiconductor as the second material to match the p-type nature of two materials [31]. Moreover, this method can be easily used to prepare CuS nanoparticle-coated composites in the presence of other nanomaterials such metal oxides. Also, this method can be used for the bulk production of CuS nanoparticle-coated composites. We have synthesized CuS nanoparticle-coated TiO_2_ composites, and XRD and EDX spectra of this composite are shown in Additional file 1: Figure S1. To the best of our knowledge, no reports have been found regarding this simple method to prepare CuS nanoparticle-coated composites.

## Methods

### Materials

Sodium thiosulfate pentahydrate (Na_2_S_2_O_3_·5H_2_O), copper(II) sulfate (CuSO_4_), triethyl amine, and ammonium thiocyanate were purchased from Sigma-Aldrich, and they were all used as received.

### Synthesis of Nano-CuS-Coated CuSCN

Triethyl amine hydrothiocyanate (THT) was synthesized as described in our previous publication [31]. 0.1 M copper sulfate (100 ml) was mixed with 0.1 M sodium thiosulfate pentahydrate (100 ml) in 1:1 ratio and stirred for 30 min. Then, different volumes of 0.1 M THT solution were added dropwise, and the resultant solution was kept overnight while stirring. The precipitate was then centrifuged and washed with distilled water several times prior to characterization.

### Characterization

The morphology of prepared NPs and nanocomposites were observed with scanning electron microscope (SEM; Hitachi SU6600) and high-resolution transmission electron microscope (HRTEM; JEOL JEM 2100). Electron energy loss spectroscopy (EELS-GATAN 963 spectrometer) was used to determine the elemental spectroscopy. Powder X-ray diffraction patterns were recorded by Bruker D-8 Focus instrument (40 kW, 40 mA) with Cu-Kα radiation with a wavelength of 0.15418 nm. UV-Vis spectra were obtained by Shimadzu UV-3600 NIR spectrometer and diffuse reflectance modes.

## Results and Discussion

**Fig. 1:**
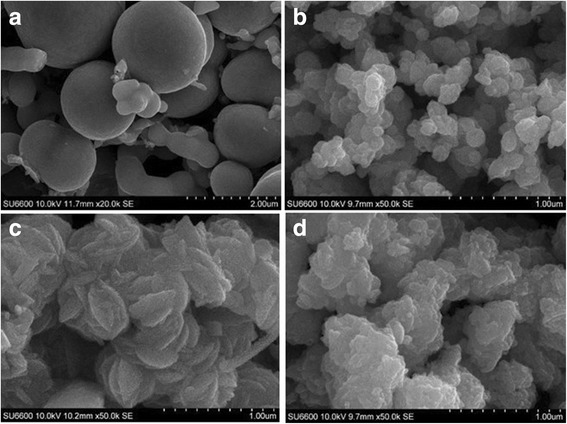
SEM pictures of **a** pure CuS, **b** CuS-coated CuSCN adding 10 ml of THT, **c** CuS-coated CuSCN adding 25 ml of THT, and **d** CuS-coated CuSCN adding 50 ml of THT

**Fig. 2:**
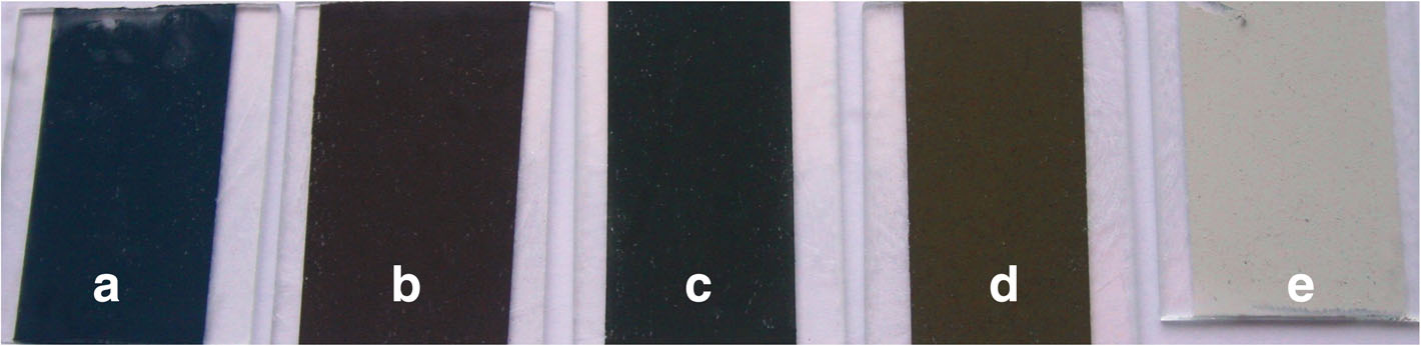
Picture of thin films: **a** CuS (0 THT), **b** CuS-coated CuSCN (10 ml THT), **c** CuS-coated CUSCN (25 ml THT), **d** CuS-coated CuSCN (50 ml-THT), and **e** CuSCN only (100 ml THT)

The mixture of copper sulfate (0.1 M–100 ml) and sodium thiosulfate (0.1 M–100 ml) in 1:1 ratio (solution A) produced a blue-colored precipitate after overnight reaction. It was noticed that the solution A was light green in color just after mixing and no precipitate was seen. The dark blue-colored precipitate was developed after overnight reaction and contained a large quantity of spherical shaped microparticles as well as small quantity of nanoparticles as shown in Fig. 1a. When THT (< 0.1 M–100 ml) was added to the solution A, white-colored CuSCN was formed immediately. The color of this mixture turned into light brown upon aging the mixture which is due to the deposition of CuS nanoparticles on the surface of CuSCN. When the volume of THT (0.1 M) varies from 0 to 100 ml in the solution A, the color of the composite after overnight reaction changed as shown in Fig. 2. These composite films were made on glass plates by doctor blade method. When 100 ml of THT is present, only gray-colored pure CuSCN was produced as shown in Fig. 2e, whereas the solution A without THT produced only dark blue-colored CuS (Fig. 2a). With the addition of 100 ml of THT into the solution A, the Cu^+^ in the solution reacts with the SCN^−^ and produced CuSCN without leaving further Cu^+^ to deposit as CuS on the CuSCN crystal. When THT varies by 10, 25, and 50 ml, three different colored composites of CuS-coated CuSCN were produced as shown in Fig. 2b–d.

Figure 1 shows the morphology of CuS (a) and CuS-coated CuSCN nanoparticles (b–d). Figure 1a has significant amount of microscale spherical particles of CuS together with scattered CuS nanoparticles. Images (b) to (d) show CuS-coated CuSCN nanoparticles where CuS cannot be distinguished from the CuSCN. The notable difference in this methodology is the in situ synthesis of CuS nanoparticles on CuSCN instead of precipitation of large spherical shaped CuS.

**Fig. 3:**
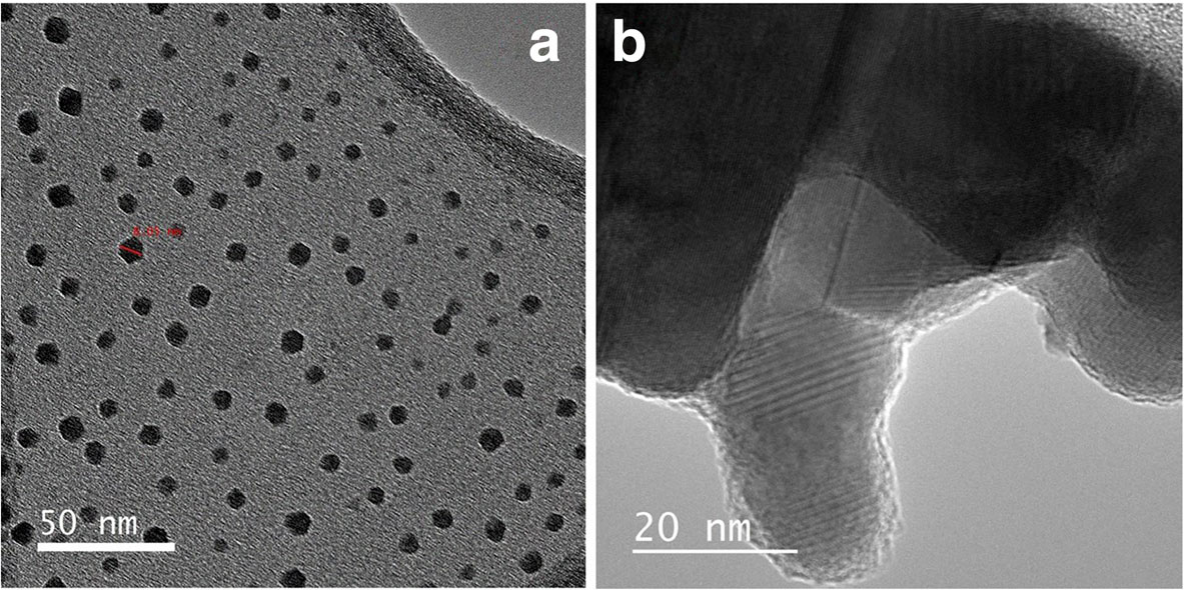
TEM images of **a** CuS nanoparticles and **b** bulk CuSCN crystals in the CuS-coated CuSCN composite (10 ml THT)

**Fig. 4:**
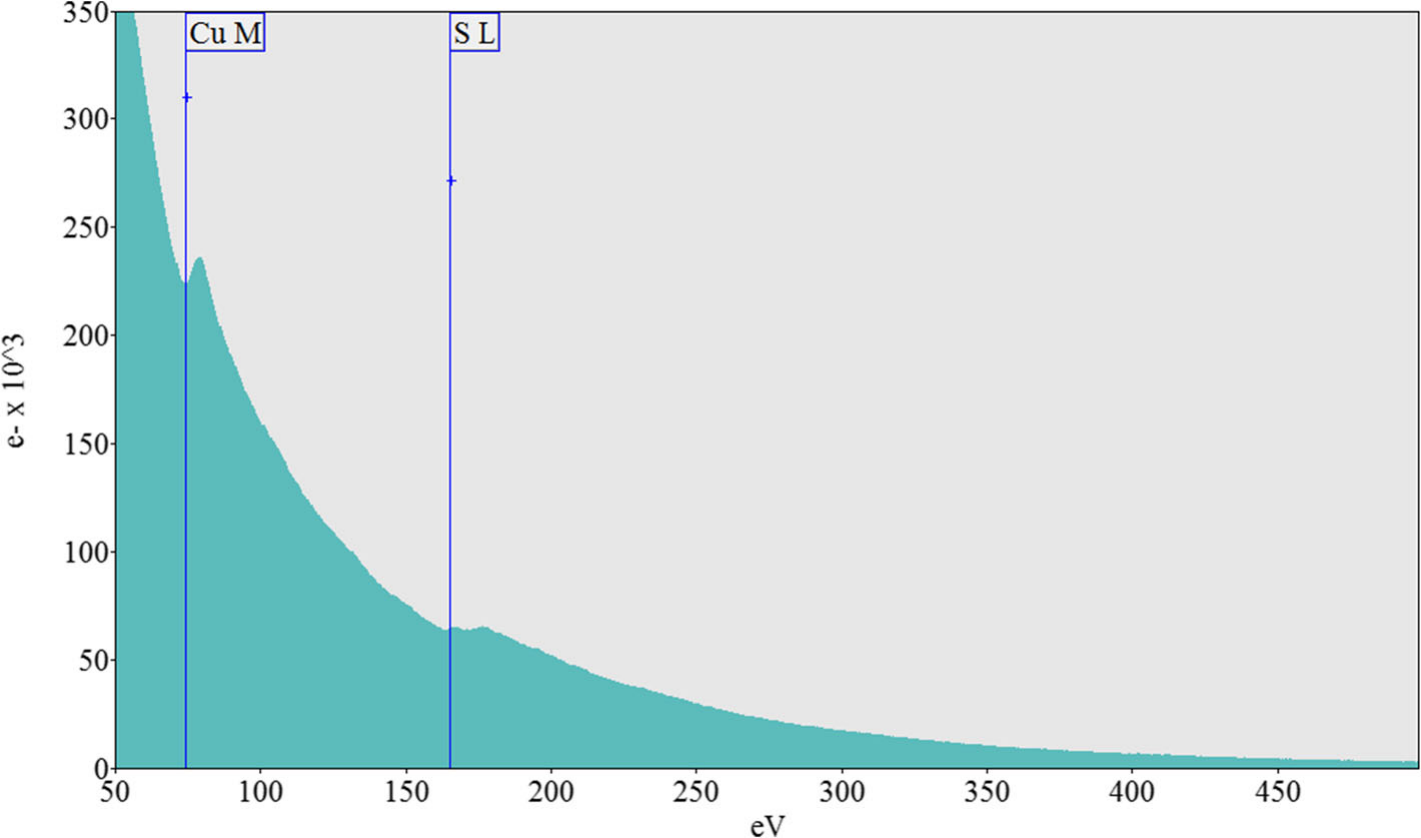
EELS spectroscopy of a hexagonal CuS nanoparticle in the CuS-coated CuSCN composite (10 ml THT)

To distinguish CuS nanoparticles from the CuSCN, TEM analysis was carried out and images are shown in Fig. 3. Distribution of CuS nanoparticles in the range of nearly 3 to 10 nm can clearly be seen in Fig. 3a, and CuSCN particle matrix is shown in Fig. 3b. It is interesting to note here that no CuS nanoparticles can be seen on the CuSCN particles after ultra-sonication of composite with ethanol solvent as shown in Fig. 3b. This separation of CuS from CuSCN matrix is taken place due to the sonication of suspension in the ethanol solution during TEM sample preparation. Before sonication, clear solution of CuS-coated CuSCN particles were obtained; however, after the sonication, colored solution appeared due to separation of CuS nanoparticles from CuSCN matrix; see Additional file 1: Figure S2. CuS nanoparticles were further investigated using electron energy loss spectroscopy (EELS) by isolating a nanoparticle in a holy-carbon TEM grid to identify the compound correctly. It was noted that only Cu (74 eV) and S (165 eV) peaks were observed while no carbon peak was observed at 284 eV, as shown in Fig. 4.

**Fig. 5:**
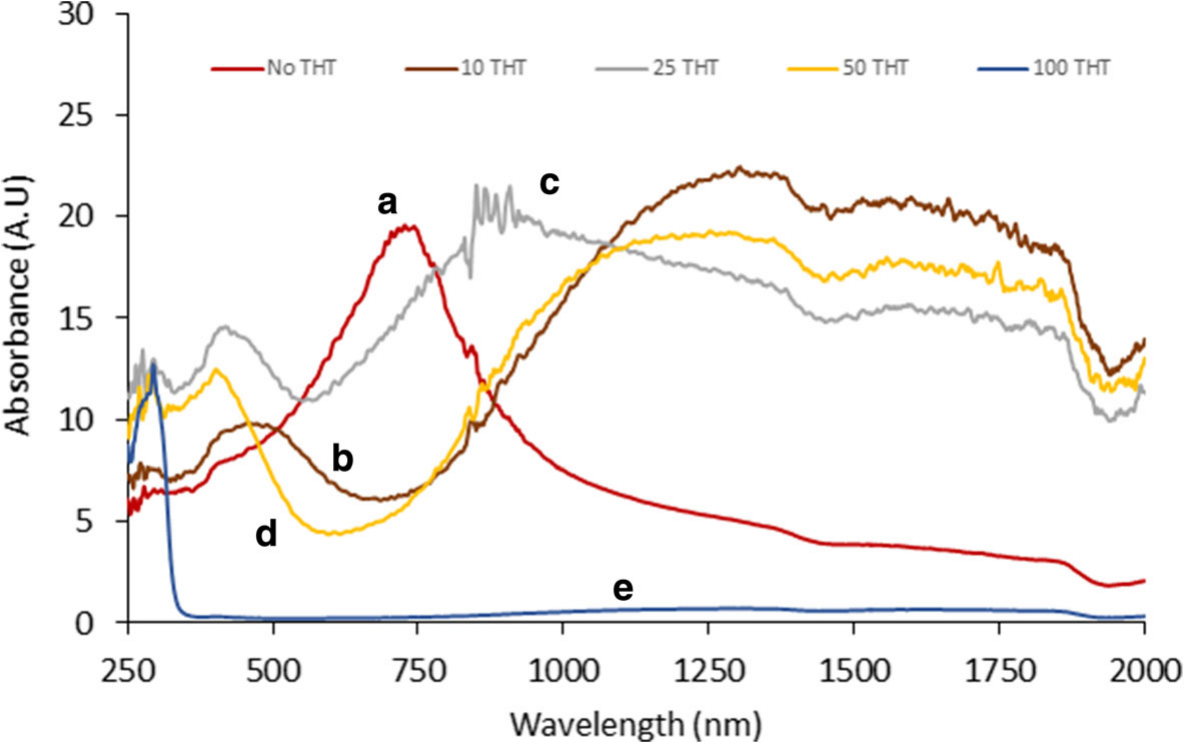
Absorbance spectra of (a) pure CuS without adding THT; (b) CuS-coated CuSCN, adding 10 ml of THT; (c) CuS-coated CuSCN, adding 25 ml of THT; (d) CuS-coated CuSCN, adding 50 ml of THT; and (e) Pure CuSCN, adding 100 ml of THT

Figure 5 (a) shows the absorption spectra of pure CuS and (b), (c), and (d) are that of CuS-coated CuSCN by adding 10, 25, and 50 ml of THT, respectively. Figure 5 (e) represents the absorption spectra of pure CuSCN by adding 100 ml of THT. It is clearly noticeable the unique absorption curve for each material in the visible and IR regions. Pure CuS has an absorption maximum around 735 nm whereas pure CuSCN has a slight absorption at IR region but almost no absorption in the visible region. It is acceptable the fact that CuSCN has no absorption in visible region since it is a high bandgap p-semiconductor (~ 3.6 eV) [31]. It is very interesting to note that CuS-coated CuSCN materials have unique properties compared to pure CuSCN and CuS. This material has absorptions in both the visible region and IR region up to 1900 nm. The brown-colored CuS/CuSCN synthesized adding 10 ml of THT (Fig. 5 (b)) has the highest absorption in the IR region coupled with another maximum absorption at 465 nm in the visible region. However, composite synthesized adding 25 ml of THT (Fig. 5 (c)) has a maximum absorption at 425 nm and a slightly attenuated IR absorption in comparison with Fig. 5 (b). It is noted that the composite synthesized adding 50 ml of THT (Fig. 5 (d)) has an intermediate IR absorption in comparison with Fig. 5 (b), (c) and maximum visible absorption at 410 nm. An increase in the amount of THT in the solution A has resulted in a blue shift of the absorption maximum in the visible region as shown in Fig. 4.

**Fig. 6:**
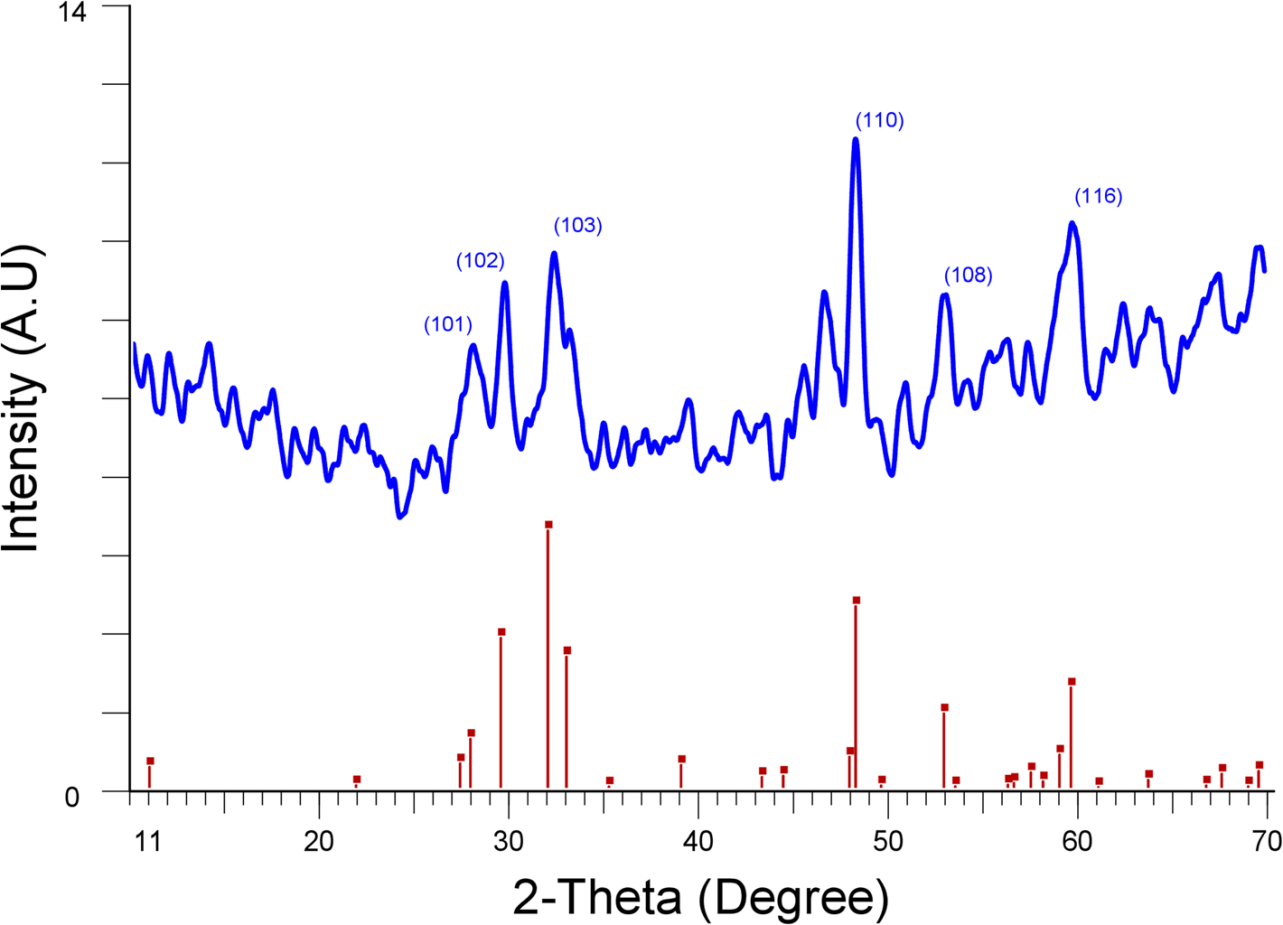
XRD spectrum of CuS prepared by mixing copper sulfate and sodium thiosulfate without adding THT

**Fig. 7:**
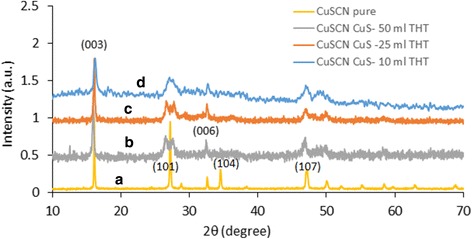
XRD spectra of (a) pure CuSCN, adding 100 ml of THT, (b) CuS-coated CuSCN adding 50 ml of THT, (c) CuS-coated CuSCN, adding 25 ml of THT, (d) CuS-coated CuSCN, adding 10 ml of THT

Figure 6 shows the XRD spectrum of dark blue-colored CuS without adding THT. This spectrum clearly matches with the standard covellite structure of CuS given at JCPDS number 03-065-3561 as depicted in Fig. 6. Figure 7 shows the XRD spectra of CuS-coated CuSCN with the addition of THT (a) 100 ml, (b) 50 ml, (c) 25 ml, and (d) 10 ml. Figure 7 (a) represents only CuSCN, and it is consistent with the β form of CuSCN data given in the JCPDS number 29-0581. Figure 7 (b)–(d) represents the XRD spectra of CuS-coated CuSCN. It is hard to distinguish the peaks of CuS from the CuSCN in the composites since most of the peaks of individuals are nearly overlapped except the peak at 16.1° of CuSCN. Splitting of peaks at near ~ 27.3 appeared from “b” to “d” spectra of Fig. 7 which may be attributed to the interaction of peaks at ~ 27.9 of CuS and 27.2 of CuSCN. On the other hand, since CuS particles are too small in the range of 3 to 10 nm as well as having week crystallization, CuS peaks may not appear intensively in the bulk of CuS-coated CuSCN material. This type of weak X-ray diffraction peaks was reported by other workers. Cruz et al. have synthesized CuS nanoparticles (13.5 ± 3.5 nm) coating on a glass substrate by chemical bath deposition technique, and they have experienced almost amorphous looking XRD pattern even when particle size was ~ 13.5 nm [32]. Nath et al. also have experienced the same, extremely weak, XRD pattern when CuS nanoparticles were deposited on glass substrates [33].

Resistivity of each sample was measured by making thin films on the Cr/Pt-sputtered glass electrode whose 1-mm gap has no metal coating. Thin films were prepared by the doctor blade method. In this method, slurry paste of compound is placed on the substrate whose nonactive area is covered with thin tape and then blade or glass rod is moved over the attached tape so as to remove the excess slurry and form uniform thin film on the essential area of the substrate. Table 1 shows the calculated resistivity values of each sample. It is interesting to note that only CuS which has almost microspherical particles shows large resistance compared to CuS-coated CuSCN which has very low resistance in ohm range. Creation of copper vacancies while deposition of CuS on CuSCN crystal might be the one reason to have lower resistivity of CuS-coated CuSCN. Size of the CuS particles may also affect the smooth interconnectivity between each particle. To see the crystallization effect on conductivity, we further annealed CuS-coated CuSCN (10 ml of THT) thin film at 250 °C for 20 min under nitrogen atmosphere. It was interesting to note that the resistance of the annealed film reduced to 5 Ω (*ρ*—0.05 Ω cm) from 15.8 Ω (*ρ*—0.16 Ω cm) before annealing. The 68% decrement of resistance at annealing under nitrogen may be attributed to the improvement of the crystallization and interconnectivity of CuS deposited on CuSCN particles (Additional file 1: Figure S3).

**Table 1:** Resistance of each thin film prepared and their calculated resistivity

Sample	Resistivity (Ω cm)	Resistance between electrodes/Ω
CuSCN only (100 ml THT)	–	Highly resistive
CuS microparticles (without THT)	–	3000
CuS-coated CuSCN (50 ml THT)	1.26	126
CuS-coated CuSCN (25 ml THT)	0.50	50.2
CuS-coated CuSCN (10 ml THT)	0.16	15.8

## Conclusions

Conductivity-tunable, different colored CuS-coated CuSCN composites were synthesized with a mixture of copper sulfate and sodium thiosulfate in the presence of THT. It was noted that CuS-coated CuSCN materials have unique properties compared to pure CuSCN and CuS. This material has absorption in both the visible region and IR region up to 1900 nm. Minimum resistivity of 0.05 Ω cm was observed for annealed (250 °C) CuS-coated CuSCN under nitrogen atmosphere. On the other hand, this method can easily be utilized to synthesize other CuS-based nanocomposite in the presence of other nanomaterials such as metal oxide.

## Additional file

Additional file 1: Facile synthesis of colored and conducting CuSCN composite coated with CuS nanoparticles. **Figure S1**. XRD and EDX spectra of CuS-coated TiO_2_. **Figure S2**. CuS-coated CuSCN composite before and after sonication in water. **Figure S3**. Resistance value of thin film prepared from CuS-coated CuSCN (adding 10 ml of THT), before and after annealing 250 °C under N_2_ atmosphere. (DOCX 1352 kb)
