# Supplementary material for: Facile Synthesis of Colored and Conducting CuSCN Composite Coated with CuS Nanoparticles
Source: Nanoscale Res Lett. 2017 Aug 23;12:507. doi: 10.1186/s11671-017-2275-6 (PMC6890889; doi:10.1186/s11671-017-2275-6)
Supplement: Supplementary file 1 — Facile synthesis of colored and conducting CuSCN composite coated with CuS nanoparticles. Figure S1. XRD and EDX spectra of CuS-coated TiO2. Figure S2. CuS-coated CuSCN composite before and after sonication in water. Figure S3. Resistance value of thin film prepared from CuS-coated CuSCN (adding 10 ml of THT), before and after annealing 250 °C under N2 atmosphere. (DOCX 1352 kb) [file 11671_2017_2275_MOESM1_ESM.docx]

**Additional file 1**

**Facile synthesis of colored and conducting CuSCN composite coated with CuS nanoparticles.**


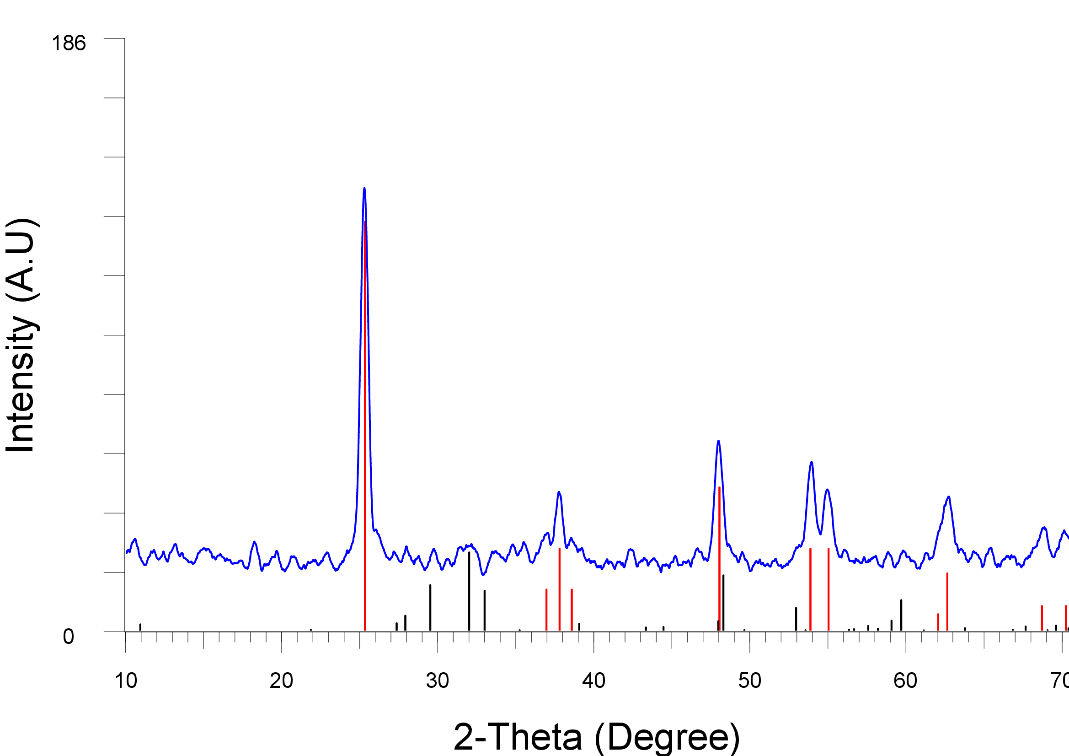


(a)

CuS (065-3561)

Anatase TiO_2_ (021-1272)

(116)

(204)

(211)

(105)

(200)

(004)

(101)


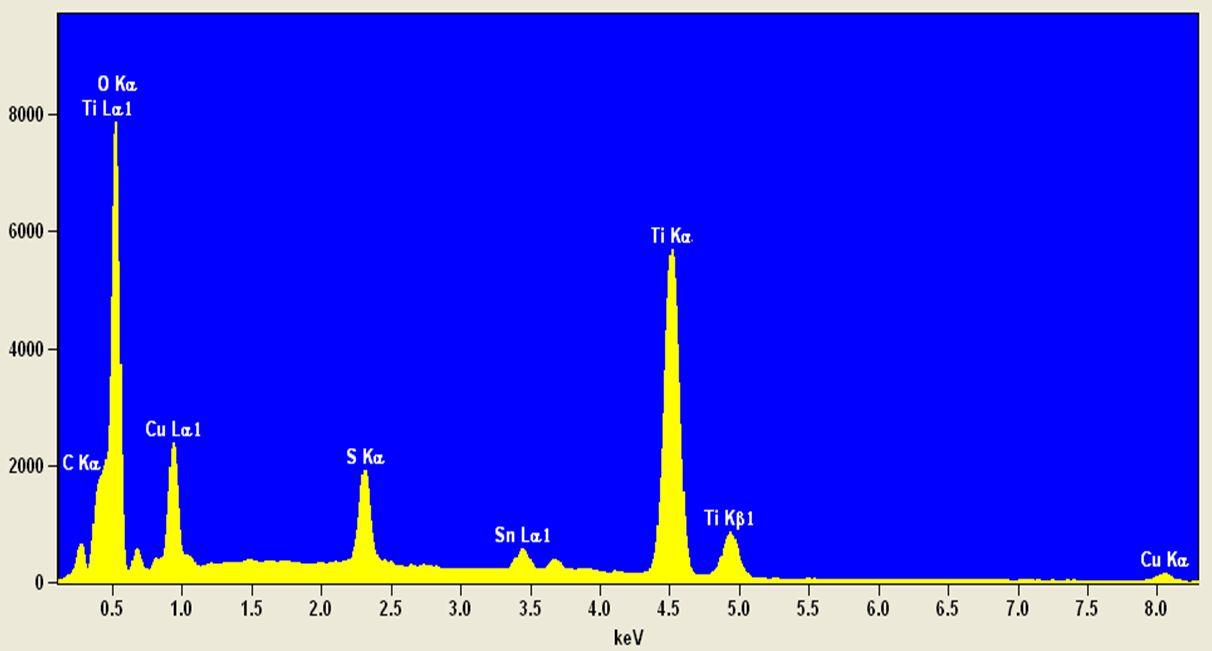


(b)

Figure 1. XRD and EDX spectra of CuS coated TiO_2_


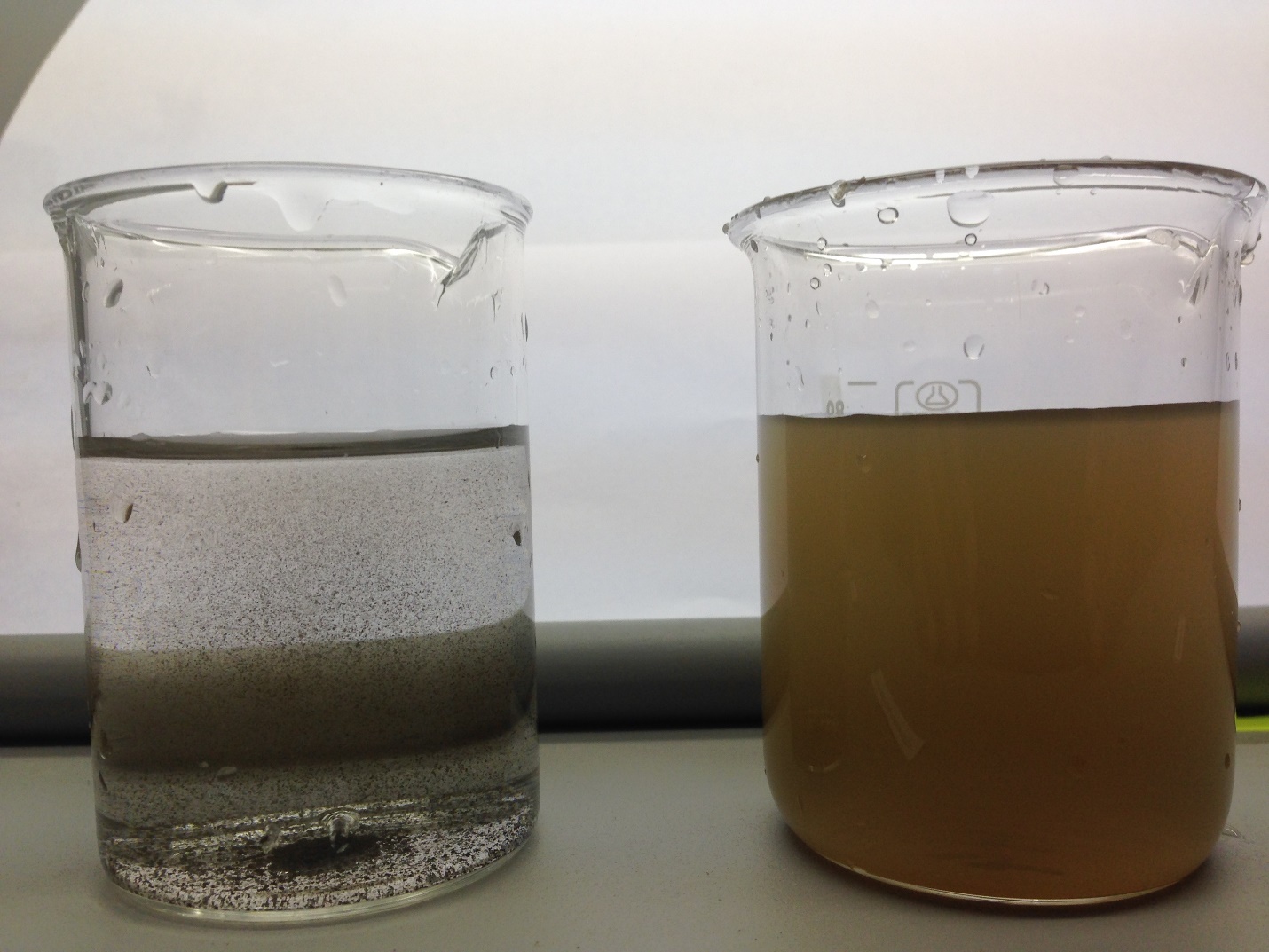


After sonication

Before sonication

Figure 2. CuS coated CuSCN composite before and after sonication in water


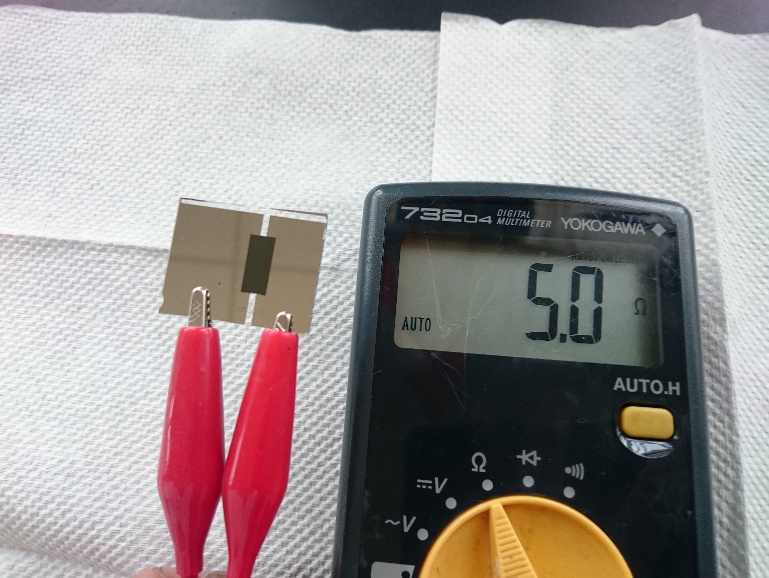

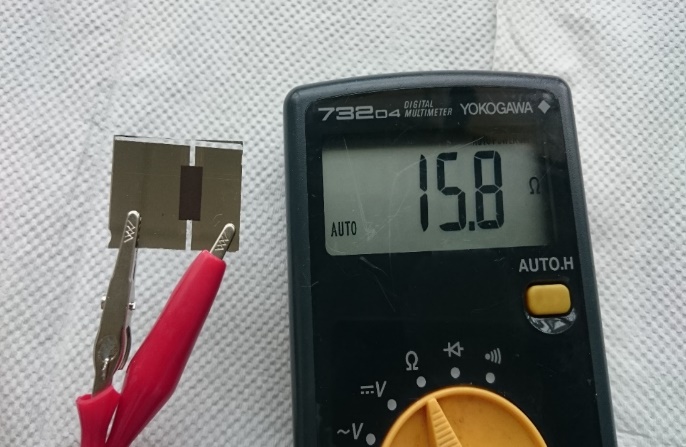

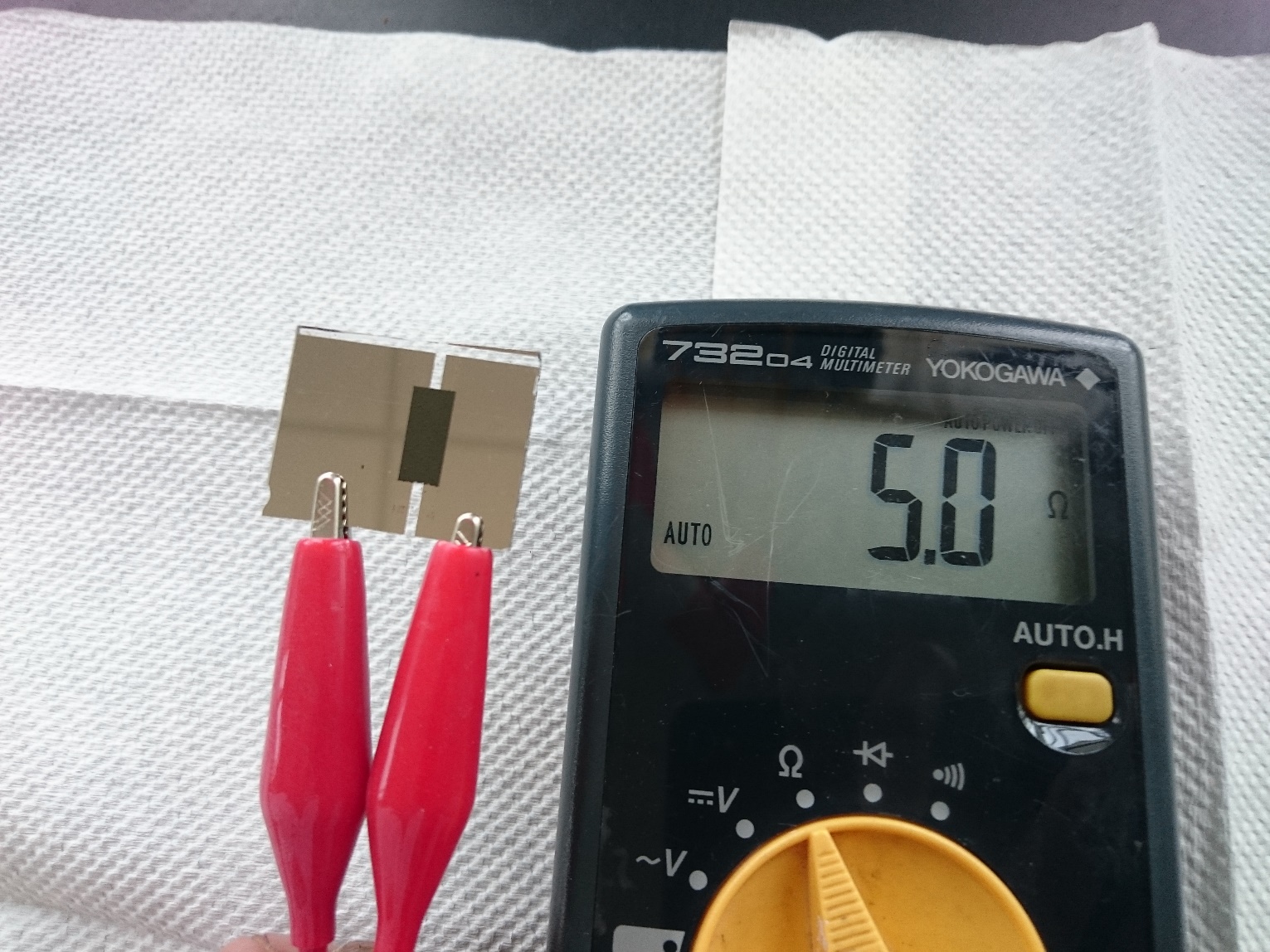


Resistivity – 0.05 Ω cm

Resistivity – 0.16 Ω cm

After heating at 250 °C under N_2_ -20 min

Before heating

10 ml THT

After heating at 250C under N2 -20 min

Resistivity – 0.05 Ω cm

Figure 3. Resistance value of thin film prepared from CuS coated CuSCN (adding 10 ml of THT), before and after annealing 250 °C under N_2_
